# Psychosocial Predictors of Weight Loss among American Indian and Alaska Native Participants in a Diabetes Prevention Translational Project

**DOI:** 10.1155/2016/1546939

**Published:** 2015-11-15

**Authors:** Edward J. Dill, Spero M. Manson, Luohua Jiang, Katherine A. Pratte, Margaret J. Gutilla, Stephanie L. Knepper, Janette Beals, Yvette Roubideaux, Special Diabetes Program for Indians Diabetes Prevention Demonstration Project

**Affiliations:** ^1^Department of Psychology, University of Colorado Denver, Denver, CO 80217, USA; ^2^Centers for American Indian and Alaska Native Health, Colorado School of Public Health, University of Colorado Denver, Aurora, CO 80045, USA; ^3^Department of Epidemiology, School of Medicine, University of California Irvine, Irvine, CA 92697, USA; ^4^Office of the Director, Indian Health Service, Rockville, MD 20852, USA

## Abstract

The association of psychosocial factors (psychological distress, coping skills, family support, trauma exposure, and spirituality) with initial weight and weight loss among American Indians and Alaska Natives (AI/ANs) in a diabetes prevention translational project was investigated. Participants (*n* = 3,135) were confirmed as prediabetic and subsequently enrolled in the Special Diabetes Program for Indians Diabetes Prevention (SDPI-DP) demonstration project implemented at 36 Indian health care programs. Measures were obtained at baseline and after completing a 16-session educational curriculum focusing on weight loss through behavioral changes. At baseline, psychological distress and negative family support were linked to greater weight, whereas cultural spirituality was correlated with lower weight. Furthermore, psychological distress and negative family support predicted less weight loss, and positive family support predicted greater weight loss, over the course of the intervention. These bivariate relationships between psychosocial factors and weight remained statistically significant within a multivariate model, after controlling for sociodemographic characteristics. Conversely, coping skills and trauma exposure were not significantly associated with baseline weight or change in weight. These findings demonstrate the influence of psychosocial factors on weight loss in AI/AN communities and have substantial implications for incorporating adjunctive intervention components.

## 1. Introduction

Although diabetes is highly prevalent worldwide, its presence among American Indians and Alaska Natives (AI/ANs) is particularly alarming [1]. Adjusting for age, AI/ANs suffer from type 2 diabetes mellitus at rates greater than two times those of non-Hispanic whites and exhibit the highest prevalence of this disease of any racial group in the United States [1, 2]. Given the sharp increase in incident diabetes among AI/ANs over the last 20 years, these circumstances seem unlikely to change without substantial intervention [3–5].

The Special Diabetes Program for Indians Diabetes Prevention (SDPI-DP) demonstration project has been implemented over the past decade to address this problem using a well-established, evidence-based preventive intervention. The SDPI-DP initiative was developed based upon the National Institute of Diabetes, Digestive and Kidney Disease's (NIDDK) Diabetes Prevention Program (DPP), which was a large-scale clinical trial that demonstrated that lifestyle interventions (e.g., changing diet and exercise habits) can be effective in delaying or preventing the onset of diabetes in individuals who are at increased risk for developing this disease [6]. The DPP outcomes did not differ significantly for various ethnic groups, including American Indians [6]; however, the DPP was conducted as a highly controlled clinical trial, which did not allow for evaluating the effectiveness of lifestyle interventions in preventing the onset of diabetes in community-based settings with underserved populations. AI/AN communities often face a lack of health care resources and a highly mobile population, thereby making it particularly difficult to implement large-scale prevention programs. Therefore, the SDPI-DP worked with experts in a variety of AI/AN communities to implement cultural adaptations to the original DPP lifestyle curriculum (e.g., the use of indigenous foods, drumming during class sessions), in order to make the program more relevant to AI/AN individuals and more transferrable to a geographically, culturally, and organizationally diverse array of settings in tribal communities [7].

The SDPI-DP demonstration program resulted in reduced diabetes incidence among high risk AI/ANs at a rate comparable to the results for AI/ANs in the original DPP study [7]. In addition, improvements in weight, blood pressure, and lipid levels were detected following the intervention [7]. However, despite the overall effectiveness with which the intervention was delivered to SDPI-DP participants, several participant characteristics were related to retention in the program; participants who were younger, were male, had less education, and had lower income were more likely not to complete the core intervention [8]. These initial findings regarding the relationship between sociodemographic factors and retention led program staff to question the potential additional impact of individual-level psychosocial factors on participant engagement, ability to grasp the knowledge conveyed, and mastery of skills related to the behavioral changes associated with the desired outcomes. Therefore, it was determined that further analyses were warranted in order to evaluate the extent to which program outcomes were related to individual-level psychosocial characteristics.

The observation of a potential impact of psychosocial factors on self-management of medical illnesses is not unique. For example, the influence of depression and anxiety on intervention outcomes for individuals with prediabetes was examined in at least one previous study, and more positive baseline mood was correlated with increased physical activity [9]. In addition, a bidirectional relationship between depression and diabetes has been previously supported. Specifically, there is evidence that diabetes may increase the likelihood of depressive episodes and that depression may increase the risk of developing diabetes [10–13]. Furthermore, psychological distress in general has also been shown to be associated with many chronic health conditions, including obesity [14, 15], which is a significant risk factor for the development of type 2 diabetes. Other studies have identified increased odds of diabetes among AI/ANs with a history of trauma and significant life stressors [16, 17].

Conversely, strong coping skills and other positive emotional attributes have been found to enhance metabolic control among those with diabetes [18]. In addition, increased spirituality has been associated with improved self-management among African Americans who suffer from diabetes [19], lower stress and higher quality of life in persons afflicted by chronic illness [20], and decreased likelihood of developing depression [21]. Although the relationship between spirituality and diabetes has not been studied specifically in AI/AN populations, previous research has highlighted the importance of religious and spiritual practices for AI/AN individuals struggling to overcome other health issues, such as the problematic use of alcohol [22]. Additionally, family support has been correlated with increased weight loss in the prevention of diabetes among Arab Americans [23]. Similarly, positive family support was correlated with improved diet in a study of older Hispanic adults with diabetes [24]. Furthermore, active family nutritional support was linked to improved control of diabetes-related factors (i.e., triglycerides, cholesterol, and HbA1c) among Navajo tribal members [25]. Finally, several psychological and behavioral factors, including increased self-efficacy, were associated with improved weight loss for DPP participants [26].

Given this prior body of evidence supporting significant relationships between a variety of psychosocial characteristics and multiple health outcomes, the correlation of psychosocial factors (psychological distress, trauma exposure, coping skills, spirituality, and family support) with a key clinical indicator of diabetes risk (weight) among AI/ANs participating in the SDPI-DP demonstration project was assessed in the present study. Resulting insights could suggest enhancements targeting such factors in the core components of SDPI-DP that hold promise for increasing its effectiveness.

## 2. Materials and Methods

### 2.1. Participants

Eligibility criteria for participating in the SDPI-DP demonstration projects were being AI/AN (based on eligibility to receive IHS services), being at least 18 years of age, and having either impaired fasting glucose (IFG) (i.e., a fasting blood glucose (FBG) level of 100–125 mg/dL and an oral glucose tolerance test (OGTT) result <200 mg/dL) or impaired glucose tolerance (IGT) (i.e., an OGTT result of 140–199 mg/dL two hours after a 75 g oral glucose load and an FBG level <126 mg/dL). Exclusion criteria included a previous diagnosis of diabetes (not including those who only have had gestational diabetes), pregnancy, end-stage renal disease on dialysis, and any condition that would affect successful participation based on provider judgment (e.g., cardiac concerns given the physical activity element of the program, severe substance use, and undergoing treatment for cancer) [7]. Participants attended a 16-session educational curriculum, a series of lifestyle coaching sessions, and community-based exercise programs focused on reducing the risk of developing type 2 diabetes through moderate weight loss, increased physical activity, and healthy eating habits.

Clinical measurements and participant surveys were obtained at baseline, within 30 days of completing the 16-session curriculum, and annually thereafter. Participants were enrolled at one of 36 tribal, Indian Health Service (IHS) or urban Indian health care programs serving 80 tribes between 2006 and 2010. Seventy-eight percent of participants were from a rural geographic setting, and 22% were from an urban area. To be included in the current study, participants minimally completed a baseline clinical assessment and a baseline survey (*n* = 3,135). The 193 individuals who completed a baseline clinical assessment but did not complete any participant surveys were excluded from these analyses. These individuals did not differ significantly from those included in the study with regard to age, gender, and baseline weight. The SDPI-DP protocol was approved by the Institutional Review Board of the University of Colorado Denver and the National IHS Institutional Review Board. When required, grantees obtained approval from other entities overseeing research in their programs (e.g., tribal review boards). All participants provided written informed consent and Health Insurance Portability and Accountability Act authorization.

A summary of participant sociodemographic characteristics is provided in Table 1. The study sample was 74% female and had a mean age at baseline of 46.7 years. Sixty-three percent of participants attended at least some college courses; 72% of participants reported annual household incomes of less than $50,000.

### 2.2. Measures

Sociodemographic variables including participant gender, age, educational status, and annual household income were collected through a survey at baseline. Participant weight was obtained at each clinical assessment using standardized procedures.

Several psychosocial variables were assessed by participant surveys at baseline and follow-up. The 6-item Kessler Distress Scale [27] included items related to general psychological distress. Frequency of participants' experience of various symptoms of depression and anxiety during the previous 30 days was assessed using this scale. Item scores ranged from 1 (none of the time) to 5 (all the time). Participants' ability to cope with life stressors was measured using the Brief Resilient Coping Scale [28]. This 4-item scale asked participants to rate descriptions of coping reactions (e.g., approaching difficult situations in creative ways, focusing on the positive growth that can come from dealing with adversity), using a scale ranging from 1 (does not describe me at all) to 5 (describes me exactly). A modified 6-item version of the Diabetes Family Behavior Checklist [29] was used to measure participants' perceptions of positive and negative family support in regard to their efforts to prevent the onset of diabetes. SDPI-DP research staff modified the original checklist slightly by removing items that referred to specific activities for individuals with diabetes (e.g., family providing suggestions about taking insulin on time) that would not have been relevant to a program focusing on diabetes* prevention*. Participants rated how often their family members provided positive support on 4 items (e.g., exercising with them) and negative support on 2 items (e.g., criticizing them for not exercising regularly). Item scores on the six items ranged from 1 (less than once a month) to 5 (at least once a day). No items were reverse-scored, as items were phrased in either a positive or negative manner, consistent with the two scored dimensions.

Two additional psychosocial variables (trauma experience and spirituality) were assessed by participant surveys only at baseline. These two particular variables were not collected at follow-up due to the expectation of their high stability across a relatively short period of time. A single dichotomous variable from a Posttraumatic Stress Disorder (PTSD) screener [30] captured whether participants had ever experienced a significant traumatic event (e.g., being the victim of a violent crime or domestic violence, being in a disaster like a flood or fire, being in combat, being seriously injured in an accident, being sexually assaulted, and witnessing someone else being seriously injured or killed). This variable was coded either 0 (no trauma) or 1 (history of trauma). Spirituality was assessed via a 7-item scale designed specifically to capture the culturally relevant components of spirituality for AI/ANs [31]; item scores ranged from 1 (strongly disagree) to 5 (strongly agree). The items on this scale were developed through consultation with tribal leaders to reflect American Indian cultural views of the connectedness of humans to all other physical and transcendental entities. The seven items were as follows: (1) I am in harmony with all living things, (2) I feel connected with other people in life, (3) I follow my tribal path, (4) when I need to return to balance, I know what to do, (5) I feel like I am living the right way, (6) I give to others and receive from them in turn, and (7) I am a person of integrity.

### 2.3. Statistical Procedures

Confirmatory factor analyses were conducted at the item level for each psychosocial scale in order to establish measurement invariance across the two time points [32, 33]. Descriptive statistics then were calculated for all psychosocial variables. Scale scores were computed as the mean of the respective items. Subsequently, latent difference scores were created to measure change over time in the outcome variable (weight) and applicable psychosocial variables [33, 34]. Latent difference scores are not subject to the restrictive assumptions of traditional ANOVA approaches and permit the measurement of change without error by including multiple indicators of each construct at each of two time points [35]. This modeling approach decomposes the data from the second time point into two components: (1) variance associated with Time 1 and (2) variance associated with the difference from Time 1. Therefore, latent difference scores allow for the estimation of baseline variance as well as variance regarding change in a construct over time.

Following these initial steps, a series of bivariate analyses were conducted within a structural equation modeling framework, which separately evaluated the relationships between each psychosocial variable and weight. For psychosocial variables that were measured at both baseline and follow-up, three parameters of primary interest were estimated: (1) the correlation between the psychosocial characteristic and weight at baseline, (2) the predictive relationship of the baseline psychosocial characteristics on change in weight, and (3) the association of change in the psychosocial characteristic with change in weight. For psychosocial variables that were measured only at baseline (i.e., trauma and spirituality), only the first two parameters were estimated.

After evaluating the bivariate relationships, a multivariate model estimated the three parameters described above simultaneously for all psychosocial variables. This model also controlled for baseline sociodemographic characteristics, including gender, age, education, and income. Psychosocial variables were eliminated from the multivariate model in a stepwise manner if they reached a *p* value greater than 0.2 for all three primary parameters, in order to arrive at a final model. Biostatisticians have suggested that a *p* value greater than 0.2 is a reasonable cutoff to eliminate variables that are clearly nonsignificant in regression models [36]. An effect size measure for the final model (*R* ^2^) was computed as the proportion of variance of change in weight that was explained by the predictor variables.

Confirmatory factor analysis and structural equation models were tested using mean and covariance structures (MACS) modeling techniques [33]. MACS analyses allow for the inclusion of mean-level information in addition to the covariance structures information of standard structural equation modeling techniques, which is necessary for the interpretation of latent difference scores. MACS analyses also provide a particular advantage over ordinary least-squares regression approaches, namely, the fact that the unreliability of instruments/scales is taken into account and that corrections are made for measurement error. When employing structural equation modeling techniques, it is important to assess the degree to which the specified model “fits” the actual data in order to determine the appropriateness of a particular model. In the present study, the Root Mean Square Error of Approximation (RMSEA_(90%  confidence  interval)_; less than .08 is adequate fit and less than .05 is good fit), the Comparative Fit Index (CFI; greater than .90 is adequate fit and greater than .95 is good fit), and the Tucker-Lewis Index (TLI; greater than .90 is adequate fit and greater than .95 is good fit) were used as indices of model fit [32]. In all models, a *p* value of <.05 was considered statistically significant.

Full information maximum likelihood (FIML) was implemented in all analyses in order to address potential bias and decreased power due to missing data [37, 38]. Furthermore, although there was very little variation across programs in class attendance for the participants included in the current study (95% of participants who completed a follow-up assessment had completed at least 14 of the 16 recommended curriculum classes), other elements of the program may have varied slightly across sites. Therefore, in order to control for the clustering of participants into 36 separate health care programs, standard errors that are robust to nonnormality and nonindependence of observations were computed using a sandwich estimator. All analyses were conducted using Mplus Version 7.11 [39].

## 3. Results

Descriptive statistics for all clinical and psychosocial variables are presented in Table 2. Between baseline and follow-up, a significant decrease of 8.58 lbs was found with regard to average weight (Δ = −8.58, *p* < .001). With regard to the change in psychosocial factors, general psychological distress decreased over time (Δ = −0.14, *p* < .001), while coping abilities increased (Δ = 0.07, *p* < .001). Furthermore, positive family support, as perceived by participants, was higher at follow-up (Δ = 0.27, *p* < .001), whereas negative family support remained stable (Δ = 0.03, *p* = .28). At baseline, 48% of SDPI-DP participants reported a lifetime history of at least one significant trauma, and the average level of reported cultural spirituality at baseline was 3.81 (on a scale from 1 to 5).

Confirmatory factor analyses of the Kessler distress, coping, and family support measures supported invariance of factor loadings and intercepts across the two measurement time points, which indicates that the measures exhibited similar structures and measured the same constructs across time. Strong factorial invariance was established for both the Kessler distress and coping measures, with all factor loadings and intercepts constrained to be equal across time. Partial measurement invariance was established for the family support measure, as one of the intercepts for the positive family support scale was not invariant across time. It is generally acceptable to use measures with partial invariance in further structural models, if at least two indicators (scale items) have an invariant factor loading and intercept [32, 33]. In the case of the family support measure, all six items exhibited loading invariance, and all but one item exhibited intercept invariance. It was important to establish that these psychosocial measures had identical or near identical structures at both time points in order to calculate reliable and valid difference scores.

Bivariate analyses were performed prior to running the multivariate model in order to evaluate the strengths of individual predictor/outcome relationships (see Table 3). All estimates (*ψ* = covariance; *β* = regression coefficient) are provided in an unstandardized metric in order to allow meaningful interpretation based upon the original scale ranges. All bivariate models exhibited good model fit (RMSEA < .05; CFI > .95; TLI > .95), and several significant correlations were found between psychosocial characteristics and weight at baseline. Greater psychological distress at baseline was related to higher baseline weight (*ψ* = 2.44, *p* < .001). In addition, greater negative family support was significantly correlated with higher baseline weight (*ψ* = 4.55, *p* < .001), whereas greater identification with culturally relevant spirituality was associated with lower baseline weight (*ψ* = −3.13, *p* < .001). Baseline levels of coping, positive family support, and trauma experience were not significantly related to baseline weight.

In addition to investigating the relationships between psychosocial characteristics and weight at baseline, regression analyses were conducted in order to elucidate the predictive relationships between psychosocial variables (independent variables) and change in weight from baseline to follow-up (dependent variable). The results of these analyses underscore the importance of psychological distress and family support in predicting weight loss in AI/ANs with prediabetes. Greater psychological distress at baseline predicted less successful weight loss between baseline and follow-up (*β* = 1.85, *p* < .001), and an increase in psychological distress between baseline and follow-up was also significantly related to less successful weight loss (*β* = 3.23, *p* < .001). Participants who reported an increase in* positive *family support after the intervention were more successful in losing weight (*β* = −3.56, *p* < .001). Conversely, higher* negative *family support at baseline as well as an increase in* negative *family support after the intervention was significantly associated with less weight loss (*β* = 1.38, *p* = .003 and *β* = 3.13, *p* < .001, resp.). Coping, trauma experience, and cultural spirituality were not significantly related to weight change.

**Figure 1:**
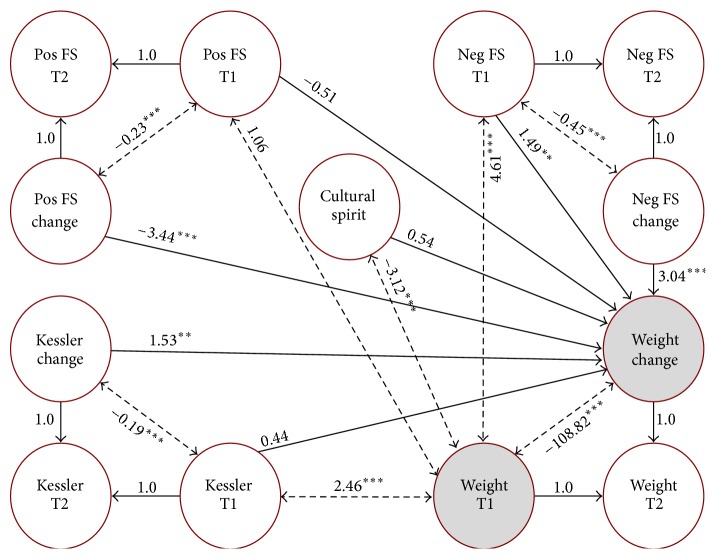
Final multivariate model results (unstandardized). Gender, age, education, and income were included in the model as covariates. Coping and trauma were dropped from the final model, because they were neither significant correlates of baseline weight nor significant predictors of change in weight. Double-headed arrows (dashed lines) represent correlations. Single-headed arrows (solid lines) represent regression paths. T1 = baseline; T2 = follow-up; Pos FS = positive family support; Neg FS = negative family support; cultural spirit = cultural spirituality. ^*∗∗*^ *p* < .01. ^*∗∗∗*^ *p* < .001.

After conducting the bivariate analyses, all of the psychosocial variables were included in a single multivariate model. Sociodemographic variables (gender, age, education status, and annual household income) that have previously been shown to be related to participant engagement and retention in the SDPI-DP program [8] were entered as covariates in the model, in order to determine the effect of the psychosocial factors on weight change above and beyond any potential effect of sociodemographic characteristics. Using the stepwise procedure described above, coping and trauma experience were dropped from the final model, as they were neither correlated with baseline weight nor predictive of weight change. Results of the final multivariate model are presented in Figure 1, which mirror the results of the bivariate models, with one exception. After controlling for sociodemographic factors and other psychosocial variables, baseline psychological distress was no longer predictive of weight loss from baseline to follow-up. However, the correlations between psychological distress, negative family support, and cultural spirituality with weight at baseline remained significant. In addition, change in psychological distress, positive family support, and negative family support over the course of the intervention, as well as levels of negative family support at baseline, remained significantly associated with change in weight. Overall, the final multivariate model exhibited good model fit (RMSEA = .026_(.025−.028)_; CFI = .966; TLI = .958) and accounted for 11% of the variability in weight change. This proportion of variance explained is not large, but it does represent a medium effect size [40].

## 4. Discussion

The importance of psychosocial characteristics as sources of diabetes risk and resilience has been demonstrated previously among AI/ANs [16, 17, 25, 41]. The present study is a critical first step in moving from research focused primarily on individuals* with diabetes* to examining factors related to successfully* preventing incident diabetes* among Native people at high risk of the disease. Although the influence of depression and anxiety on intervention outcomes for prediabetic individuals was examined in at least one previous study [9], the present study is the first to focus on determining which psychosocial factors successfully predict a specific outcome of a large-scale initiative aimed at preventing the onset of diabetes in the AI/AN population. Moreover, the statistical approach employed in the current study made it possible to simultaneously examine the relative contributions of the various psychosocial factors to successful health changes in a single model, unlike previous studies that have analyzed psychosocial factors in isolation of one another.

Specifically, structural equation modeling provides the ability to simultaneously examine the relationships of the psychosocial variables to a key clinical outcome with regard to baseline levels and change over time. As expected, when analyzing such relationships in a bivariate manner, several psychosocial factors were related to baseline levels of weight. Higher levels of psychological distress and negative family support were associated with higher weight, whereas greater spirituality was correlated with lower weight. The same pattern of correlations of these three psychosocial variables with weight at baseline also was supported in the multivariate model when controlling for sociodemographic factors.

Psychosocial factors were also related to the degree of weight change following participants' completion of the SDPI-DP intervention. Greater psychological distress at baseline and increased psychological distress over the course of the intervention both contributed to less weight loss. Similarly, greater negative family support at baseline and increased negative family support over the course of the intervention were associated with a smaller reduction in weight. Increased positive family support, on the other hand, predicted greater weight loss. Controlling for sociodemographic factors within a multivariate model, change in psychological distress, negative family support, and positive family support, as well as baseline levels of negative family support, continued to significantly affect weight reduction. The results of the present study are consistent with prior research on psychological distress as a risk factor with regard to chronic illness [14, 15, 18] and with previous findings regarding the role of positive family support in both reducing the risk of and successfully managing diabetes [23–25].

The results underscore the importance of regularly assessing the psychosocial status and functioning of AI/ANs at high risk of diabetes. Prevention programs will be well served by developing the capacity to evaluate and monitor participants' mental health status, including the presence of depression and anxiety, the nature and extent of their spirituality, and the adequacy of their family support. These personalized assessments, combined with the knowledge of the general effects of psychosocial factors uncovered in the present study, will allow program staff to know which adjunctive interventions may maximize participant benefit with respect to the desired outcomes (e.g., weight loss). For example, by increasing the focus on mental health components within the core curriculum, one could strengthen participants' strategies for decreasing depressive and stress-related symptoms, which then may make it more likely that the participants will be more engaged in the intervention and experience more successful weight loss. Offering self-management techniques, simple cognitive-behavioral skills, and referral to local support groups or treatment options is a logical extension of the goals, process, and structure of an intensive lifestyle balance intervention. Additionally, knowing that a participant has a strong preexisting spiritual focus may be helpful information for program staff who then may be able to use a participant's connectedness to the natural world as a pathway to increase motivation to engage in a healthier lifestyle. Likewise, given the strong relationship between family support and program outcomes, a greater effort should be made to incorporate close family members into various aspects of the prevention program. SDPI-DP demonstration projects have begun to do so, guided by their initial impressions of the potential gains. For example, some programs encourage participants to identify a support person to attend curriculum classes and other program-related activities with the participant.

The present study has several limitations, which suggest directions for future research. Data specific to the psychosocial characteristics were collected solely by self-report, thereby possibly increasing shared method variance and artificially strengthening the relationships among these variables. Future studies may benefit from using a variety of methods to operationalize and assess similar constructs. For example, family support could be measured through multiple informants, including close family members, and levels of depression and anxiety could be assessed through interview-based rating scales. Nevertheless, the primary relationships of interest were between self-reported psychosocial characteristics and an objective clinical measure (weight), which were not subject to problems of shared method variance. It also bears noting that trends over only two time points were analyzed. The relationships between psychosocial factors and program outcomes may wax and wane over a longer follow-up period, or certain interactions may occur over time that are not evident within a relatively short follow-up period.

In addition, SDPI-DP participants were more likely to be female, be older, have a higher level of education, and have a higher household income than the general AI/AN adult population [42]. Though previous research has shown similar trends when comparing clinical populations to the general population [43, 44], the generalizability of the present findings to individuals with widely differing sociodemographic backgrounds may be limited. For example, it is possible that weight change for males may not be as strongly related to psychological distress or family support as it was for this largely female sample. In addition, individuals with less education and lower household income than the participants in the current study may be more likely to have suffered a greater number of significant traumas. Future studies should attempt to enroll individuals with broader sociodemographic characteristics and should include a measure of the number of traumas experienced, which would provide the opportunity to analyze a possible additive effect of repeated trauma upon successful weight loss that was not possible with the dichotomous trauma item used in the present investigation. Similarly, although both rural and urban participants were included in the study, the majority of participants lived in rural settings, which may further limit the generalizability of the results. Although it would be difficult to extend these results to the mainstream American population without further research, the current findings may also be applicable to other populations that share similar structures and values with AI/AN communities (e.g., a greater emphasis on extended family support as opposed to individualism; a spiritual emphasis on connectedness to others and nature).

Furthermore, although a medium effect size for the prediction of weight change within the multivariate model was observed (11% of outcome variability explained), additional factors are likely at work and will need to be addressed to more comprehensively improve the effectiveness of such prevention programs. Some additional factors may include lack of access to healthy food selections, high levels of family and caregiver stress that make it difficult to follow through with healthy eating and exercise routines, and lack of transportation to attend program classes. Moreover, characteristics of the treatment team and health care program in general previously have been shown to be related to participant retention, which in turn predicts program outcomes [7, 8]. Therefore, it likely will be critical to incorporate a multifaceted approach to crafting additional components that promise to enhance the intervention. For example, rather than just adding a stand-alone mental health screening module, a program might consider addressing barriers to participation (e.g., lack of transportation) in concert with increasing positive family support and thereby decrease the isolation that can lead to psychological distress. Finally, a more precise and comprehensive assessment of mental health status would enable a program to determine the most appropriate approach for decreasing symptoms likely to interfere with participation in the preventive intervention. In light of the relationship between depression and diabetes [10–13, 45, 46], referral to a mental health professional is a logical option to be pursued, although other possibilities, such as a group treatment model, should also be considered given the limited numbers of mental health providers within tribal, IHS, and urban Indian health care programs.

## 5. Conclusions

The present study demonstrates the importance of psychosocial factors for maximizing the potential benefits to participants in preventive interventions such as the SDPI-DP demonstration project. The challenge now becomes how to incorporate the lessons learned into the fabric of these programs. Augmentation of the current intervention may be achieved either directly by incorporating adjunctive components or indirectly through referral to relevant local resources. The overall goal of these program additions would be to maximize participants' engagement, their ability to grasp the knowledge conveyed, and their mastery of the skills related to the behavioral changes associated with the desired outcomes.

## Figures and Tables

**Table 1:** Characteristics of SDPI-DP participants.

Variable	M (SD) or *n* (%)
Gender	
Female	2330 (74.3%)
Male	805 (25.7%)
Age (years)	46.7 (12.6)
Education status^a^	
<High school	449 (15.2%)
High school graduate	641 (21.7%)
Some college	1330 (45.0%)
≥College graduate	538 (18.2%)
Annual household income^a^	
<$15,000	539 (21.4%)
$15,000 to <$30,000	551 (21.9%)
$30,000 to <$50,000	721 (28.6%)
≥$50,000	706 (28.0%)

*Note*. *N* = 3,135. Percentages for categorical variables do not always sum to 100% due to rounding error.

^a^Education status and annual household income were not available for all participants; therefore, *n*'s for these variables do not sum to 3,135.

**Table 2:** Correlations, means, and standard deviations at baseline and follow-up.

Variable	Weight T1 *n* = 3,135	Weight T2 *n* = 2,259	Kessler T1 *n* = 3,053	Kessler T2 *n* = 2,008	Coping T1 *n* = 3,045	Coping T2 *n* = 1,997	Pos FS T1 *n* = 2,835	Pos FS T2 *n* = 1,974	Neg FS T1 *n* = 2,742	Neg FS T2 *n* = 1,874	Trauma T1 *n* = 2,896	CultSpir T1 *n* = 2,905
Weight T1	—											
Weight T2	.98^*∗∗∗*^	—										
Kessler T1	.07^*∗∗∗*^	.08^*∗∗∗*^	—									
Kessler T2	.08^*∗∗∗*^	.10^*∗∗∗*^	.67^*∗∗∗*^	—								
Coping T1	−.03	−.02	−.20^*∗∗∗*^	−.17^*∗∗∗*^	—							
Coping T2	−.04	−.04	−.17^*∗∗∗*^	−.25^*∗∗∗*^	.54^*∗∗∗*^	—						
Pos FS T1	.02	.04	−.03	−.05	.16^*∗∗∗*^	.11^*∗∗*^	—					
Pos FS T2	.05	.03	−.06	−.16^*∗∗∗*^	.10^*∗∗*^	.17^*∗∗∗*^	.49^*∗∗∗*^	—				
Neg FS T1	.10^*∗∗∗*^	.11^*∗∗∗*^	.17^*∗∗∗*^	.11^*∗∗*^	−.07^*∗*^	−.04	.58^*∗∗∗*^	.19^*∗∗∗*^	—			
Neg FS T2	.08^*∗*^	.12^*∗∗*^	.15^*∗∗∗*^	.19^*∗∗∗*^	−.06	−.08^*∗∗*^	.28^*∗∗∗*^	.37^*∗∗∗*^	.45^*∗∗∗*^	—		
Trauma T1	.02	.02	.23^*∗∗∗*^	.16^*∗∗∗*^	.09^*∗∗∗*^	.08^*∗∗*^	−.02	−.04	−.00	.04	—	
CultSpir T1	−.11^*∗∗∗*^	−.11^*∗∗∗*^	−.25^*∗∗∗*^	−.17^*∗∗∗*^	.37^*∗∗∗*^	.31^*∗∗∗*^	.17^*∗∗∗*^	.13^*∗∗∗*^	−.07^*∗∗*^	−.05	−.01	—
M	217.80	209.22^*∗∗∗*^	1.72	1.58^*∗∗∗*^	3.34	3.41^*∗∗∗*^	2.32	2.59^*∗∗∗*^	1.66	1.69	0.48	3.81
SD	52.35	50.88	0.66	0.55	0.65	0.64	0.70	0.77	0.89	0.86	0.50	0.55

*Note*. T1 = baseline; T2 = follow-up; Pos FS = positive family support; Neg FS = negative family support; CultSpir = cultural spirituality. Weight was measured in pounds. Asterisks beside a follow-up mean indicate a significant difference from the corresponding baseline mean.

^*∗*^ *p* < .05. ^*∗∗*^ *p* < .01. ^*∗∗∗*^ *p* < .001.

**Table 3:** Correlates of baseline weight (*ψ*) and predictors of change in weight from baseline to follow-up (*β*) (unstandardized bivariate model results).

Psychosocial variable	Correlation of weight and psychosocial variable at baseline			Prediction of change in weight by psychosocial variable at baseline			Association of change in weight with change in psychosocial variable		
	*ψ*	SE	*p*	*β*	SE	*p*	*β*	SE	*p*
Kessler distress	2.44	0.63	<.001	1.85	0.43	<.001	3.23	0.50	<.001
Coping	−0.84	0.90	.347	0.11	0.41	.796	−0.59	0.55	.285
Positive family support	0.92	0.87	.290	−0.36	0.61	.559	−3.56	0.59	<.001
Negative family support	4.55	1.12	<.001	1.38	0.46	.003	3.13	0.55	<.001
Trauma	0.41	0.67	.540	0.73	0.57	.199			
Cultural spirituality	−3.13	0.67	<.001	0.36	0.37	.326			

*Note*. Positive family support and negative family support were tested within the same model, because they are two factors of one measure. Trauma and cultural spirituality were measured only at baseline.
